# An interactive web-based tool for computing BMI standard deviation scores in Italian children and adolescents using national growth references

**DOI:** 10.1007/s40519-026-01887-9

**Published:** 2026-07-03

**Authors:** Livio Tarchi, Elena Mattesini, Cristiano Dani, Eleonora D’Areglia, Giovanni Castellini, Valdo Ricca

**Affiliations:** 1 Department of Neuroscience, Psychology, Drug Research and Child Health, University of Florence, Viale Pieraccini, 6, Florence, FI Italy; 2 Department of Health Sciences, University of Florence, Florence, FI Italy; 3 University Hospital of Florence, Florence, FI Italy

**Keywords:** BMI z-score, Weight, Obesity, Growth curve, Anorexia, Underweight, Overweight

## Abstract

**Background:**

Body mass index (BMI) standard deviation scores (SDS) are essential for age- and sex-adjusted assessments in pediatric populations, including growth trajectories. Their accuracy depends on the use of population-specific reference data, as BMI distributions vary substantially across countries. Despite the availability of internationally recognized growth standards, no accessible computational tool tailored to Italian normative data has been previously described.

**Objectives:**

This paper describes the development and validation of an interactive, freely accessible web-based calculator for deriving BMI-SDS in Italian children and adolescents, based on the nationally representative Cacciari et al. (2006) growth reference dataset.

**Methods:**

BMI-SDS computation is based on the LMS parameterization of Italian reference curves, which provide sex-specific L (skewness), M (median), and S (coefficient of variation) values at half-year age intervals from 2 to 20 years for a nationally representative Italian sample of approximately 70,000 children and adolescents.

**Results:**

The tool was implemented in R using the Shiny web application framework and is hosted at https://turius.shinyapps.io/BMI_SDS_IT/. Given user-entered weight, height, age, and sex, the tool computes BMI (kg/m^2^), derives the corresponding BMI-SDS and returns i) age- and sex-adjusted BMI-SDS; ii) the corresponding percentile; iii) a graphical representation of the individual's position on the reference distribution.

**Conclusions:**

The tool offers a straightforward, clinician- and researcher-friendly interface for computing age- and sex-standardized BMI scores using Italian-specific references, supporting more accurate weight-status classification in both clinical practice and epidemiological research.

## Introduction

Body mass index (BMI) is a widely used index to assess weight and developmental trajectories in children and adolescents [1]. Unlike in adults, where fixed cut-offs are applied, BMI interpretation in children and adolescents requires age- and sex-specific standardization to account for the dynamic and non-linear changes in body composition that characterize normal growth trajectories [1]. This standardization is conventionally achieved through reference curves established in representative samples, enabling the derivation of BMI standard deviation scores (SDS), which allow meaningful comparison across ages and between sexes [1].

BMI-SDS provides a normalized, continuous metric with well-established properties, offering key advantages over crude percentile classification for clinical decision-making (e.g., diagnostic criteria and/or severity threshold for eating disorders, including anorexia nervosa) [2], longitudinal growth monitoring, and cross-study comparability [3]. Nevertheless, the accuracy and clinical utility of BMI-SDS critically depend on the appropriateness of the underlying reference population. Substantial evidence indicates that BMI distributions vary across countries as a function of genetic predisposition, socioeconomic conditions, environmental exposures, and cultural factors—including dietary patterns and physical activity norms. As a consequence, applying internationally derived reference curves to populations with distinct growth trajectories may introduce systematic misclassification of weight status, with implications for both individual patient management and public health surveillance [3–6].

Population-specific BMI references and corresponding computational tools have been developed in several countries. In the United States, the Centers for Disease Control and Prevention (CDC) growth charts constitute the standard reference for BMI percentile and SDS computation across pediatric age groups, with easily available online tools for computation and visualization [3]. The World Health Organization (WHO) has also published international growth standards and references, widely used in low- and middle-income country contexts [5]. Comparable online tools have also been developed for other nations [4, 6], each reflecting the growth characteristics of their respective populations.

For the Italian pediatric population, Cacciari and colleagues [1] published a comprehensive set of BMI reference curves based on a nationally representative cross-sectional sample, providing age- and sex-specific centiles for children and adolescents aged 2 to 20 years. This reference dataset comprised approximately 70,000 children and adolescents attending infant, primary, and secondary schools across Italy, recruited between 1994 and 2004 [1]. The geographic and age-by-sex distribution of the sample was designed to approximate that of the Italian school population during the last decade of the twentieth century. These data represent the most widely cited and methodologically rigorous Italian-specific growth reference currently available. However, despite the recognized clinical relevance of population-appropriate standardization, no accessible, standardized computational tool implementing these Italian references has been previously described in the literature. Clinicians and researchers working with Italian pediatric samples must currently rely on visual thresholds on growth curves [1], non-standardized spreadsheets, reference tools from different countries, or proprietary (yet free) software developed for other purposes (i.e., use by healthcare professionals after registration) [7].

Large differences in BMI growth pattern emerge between Cacciari, CDC and UK references values [1]. In particular, Cacciari described how BMI distribution is more skewed during childhood and adolescence for the Italian population. At the end of growth (here, 20 years old), median values are similar, but the 97th centiles diverge as CDC charts are much higher and increase more steeply than that of Cacciari, which on the contrary reach a plateau [1]. Given the relevance of using population-specific normative data, this brief report introduces an interactive, freely accessible web-based calculator designed to compute Italian BMI-SDS scores.

## Materials and methods

### Statistical methods

Centiles can be expressed in terms of three smooth, age-specific curves: L (skewness of the BMI distribution), M (the median BMI at each age), and S (the coefficient of variation of BMI at each age). This LMS parameterization, in tabular form, was retrieved for girls and boys separately, at half-year age intervals from 2.0 to 20.0 years, for the national (whole Italy) sample. Given an observed BMI value and the corresponding age- and sex-specific L, M, and S parameters, the BMI-SDS (z-score) is computed as:

$$SDS = \frac{ {(BMI / M)}^{L} - 1}{L * S},$$

where BMI is the observed value in kg/m^2^, and L, M, and S are the parameters tabulated for the relevant age and sex. Because the reference LMS tables are published at discrete half-year intervals, age-specific LMS values for individuals whose exact decimal age falls between two consecutive tabulated points are computed to the closest value.

### Software development

Software implementation was led by the first author (LT), who developed computational algorithms, integrated the LMS reference parameters from Cacciari et al. designed the user interface, and managed deployment of the web application. The development followed an iterative process involving retrieval of LMS parameters, the creation of structured reference tables, and the implementation of the BMI-SDS calculation algorithm in R (version 4.5.2) [8]. Then, the web interface was designed using the Shinyapp framework [9]. To facilitate use and accessibility, a dashboard-style interface was built using the shinydashboard package. Data manipulation and BMI-SDS computation were handled via the tidyverse suite of packages [10].

Internal validation was performed by comparing software-generated BMI-SDS values with manual calculations based on the published LMS equations and reference tables. Real-time input validation was implemented through shinyFeedback—which also issues an inline warning when the entered age exceeds the lower or upper boundary of the reference range (2 and 20 years, respectively).

No individual-level data are collected, transmitted, or stored by the application. All computations are performed client-side within the active session and no input values are retained after the session ends.

## Results

To facilitate adoption by clinicians and researchers, the application is freely accessible through a public web interface (https://turius.shinyapps.io/BMI_SDS_IT) and its source code is available through a public GitHub repository (https://github.com/Turius/BMI_SDS_IT). Dissemination will be supported through presentations at scientific meetings, institutional communication channels, and professional societies (e.g., societies in the field of pediatrics, endocrinology, nutrition, and mental health), as well as networks of allied healthcare professionals. The open-source nature of the project is intended to encourage visibility, reuse, and future development by the broader scientific community.

**Fig. 1:**
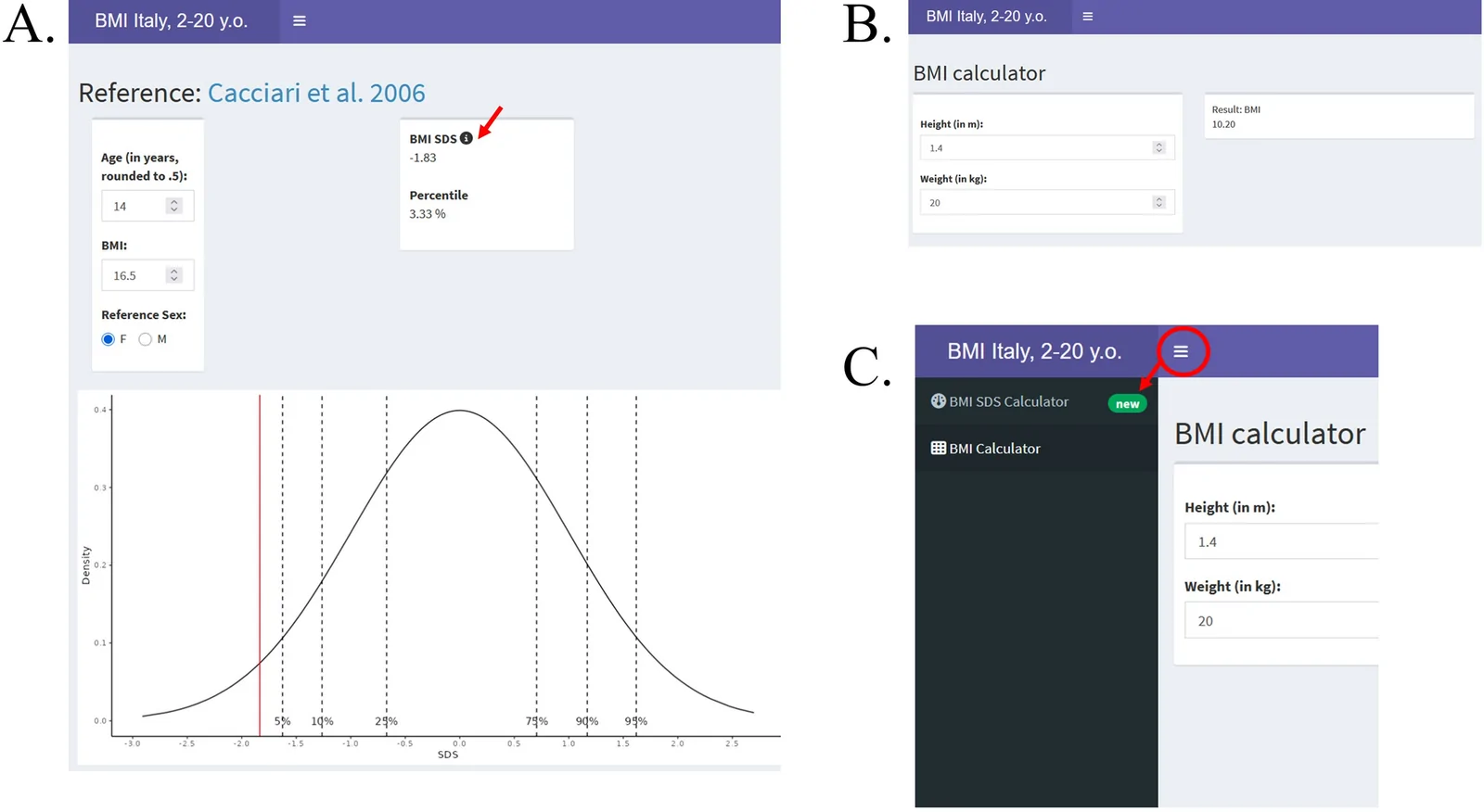
Illustrations of the interface of the BMI-SDS Italy (2–20 y.o.) web-based calculator. **A** BMI-SDS calculator tab, showing the input panel (age in years rounded to 0.5, BMI in kg/m^2^, and reference sex) alongside the output panel (computed BMI-SDS, age- and sex-adjusted percentile). The lower panel shows the standard normal density curve with dashed vertical lines marking key percentile thresholds (5th, 10th, 25th, 75th, 90th, 95th) and a red vertical line indicating the individual's position on the distribution. The red arrow points to an info icon, which shows the following text upon hovering: “BMI SDS expresses how far a child's BMI is from the age- and sex-specific references (in standard deviations—SDS). A value close to 0 is near the reference mean. A positive value indicates a BMI above the reference mean. A negative value indicates a BMI below it.” **B** The auxiliary BMI calculator tab, which derives BMI from entered height (in meters) and weight (in kilograms), providing a convenient upstream step for users who do not have BMI pre-calculated. **C** The collapsible sidebar navigation menu (accessible via the highlighted icon), showing the two available tabs: BMI-SDS calculator and BMI calculator

The user interface has two panels. The first is the BMI-SDS calculator. This tool (Fig. 1, Panel A) accepts three inputs: BMI (kg/m^2^), age (years), and a binary reference sex selector. The corresponding BMI-SDS is then derived by matching the entered age and sex to the appropriate row of coefficients, applying the respective transformation (see Statistical methods). It should be noted that, in the current implementation, age is matched to the nearest half-year entry rather than by continuous linear interpolation between adjacent entries. In addition to the numerical BMI-SDS output, the tool displays the corresponding age- and sex-adjusted BMI percentile (derived from the standard normal cumulative distribution function) and renders an interactive density plot of the standard normal distribution with a vertical marker indicating the individual's position, facilitating visual communication of the result to clinicians and patients alike.

The second tool is a simple BMI calculator for reference (Fig. 1, Panel B). The user may switch between the two through an adaptive side menu (Fig. 1, Panel C).

### Illustrative examples

Consider a 14-year-old girl with a BMI of 16.5 kg/m^2^. The tool estimates a BMI-SDS of -1.83, along with the corresponding percentile of 3.33% (Fig. 1, Panel A). This indicates that the individual's BMI is below the age- and sex-specific population average and may warrant further clinical assessment depending on the clinical context. Instead, online computation on CDC normative values would report a percentile of 11%, above either the usual clinical cut-off of 10% or 5% often used as an indicator of anorexia nervosa. 

As a second example, a 15-year-old boy with a BMI of 23.5 kg/m^2^ receives a BMI-SDS of 0.65 and a percentile of 74.17%, indicating a BMI in line with the population average for age and sex. Instead, online computation on CDC normative values would report a percentile of 85%, indicating overweight.

## Discussion

The present study describes the development and validation of a freely accessible, interactive web-based tool for computing BMI-SDS in Italian children and adolescents, leveraging Italian normative references [1]. To our knowledge, this represents the first openly available computational implementation of Italian normative BMI scores, addressing a practical gap that has persisted in Italian pediatric clinical and research practice.

The combination of numerical and graphical output supports the rapid adoption of the tool both in clinical and research practice, allowing for a precise quantification and intuitive communication of BMI-SDS/percentiles. The inclusion of an integrated BMI calculator further reduces the number of preparatory steps required, lowering the barrier to use in routine clinical settings.

As previously mentioned, the present tool fills the gap of widely accessible tools for BMI-SDS computation and visualization for the Italian population [4–6]. Its open-source architecture will hopefully facilitate future adaptation (e.g., incorporating updated Italian normative data should these become available), while also allowing the development of similar tools for other anthropometric indices (e.g., weight-for-height or waist-circumference SDS).

### Strength and limits

The tool provides a methodologically rigorous and practically accessible resource for pediatric weight-status assessment, filling a gap in the availability of nationally calibrated computational tools for the Italian clinical and research community. Moreover, BMI-SDS calculations require only age, sex, and anthropometric measurements. As all computations are performed within the active user session, privacy concerns are minimized.

The extent to which the current reference accurately reflects the contemporary Italian pediatric population is uncertain, and users should interpret SDS values accordingly. Children of immigrant background were explicitly excluded from the original reference sample. The tool may therefore be less appropriate for standardizing BMI in children with non-Italian ethnic heritage, for whom LMS parameters may not be representative [1].

A further limitation relates to the age of the underlying reference dataset. Growth references were derived from data collected between 1994 and 2004 and therefore may not fully reflect the characteristics of the contemporary Italian pediatric population [1]. BMI-SDS generated by the present tool should be interpreted in the context of these historical reference standards. The application will be updated should more recent nationally representative Italian growth references become available.

Moreover, in the current implementation, age is matched to the nearest tabulated half-year entry rather than interpolated continuously between adjacent entries, yielding an effective age resolution of 0.5 years. For children whose exact age falls midway between two entries, this introduces a small but systematic rounding artifact in the computed SDS.

### What is already known on this subject?

BMI interpretation in children and adolescents requires age- and sex-specific standardization. Italian normative scores have already been described [1], and proprietary software developed for use by healthcare professionals [7].

### What this study adds?

We present the first openly available, open-source, implementation of Italian normative BMI scores. The developed tool does not require installation or registration by users. The tool may address the need for streamlined computation of BMI-SDS in clinical or research practice.

## Conclusions

This brief report introduces an open-access, interactive tool for computing age- and sex-standardized BMI-SDS in Italian children and adolescents using national normative reference scores. Its adoption may improve the accuracy and comparability of adiposity assessment across clinical practice, epidemiological surveillance, and intervention research in Italy. Future work should consider extending the tool's functionality to additional anthropometric indices.

## Data Availability

The application is freely accessible online at (https:/turius.shinyapps.io/BMI_SDS_IT). The full source code is publicly available under an open-source license at (https:/github.com/Turius/BMI_SDS_IT) (CC-BY 4.0).
